# Different Fruit-Specific Promoters Drive *AtMYB12* Expression to Improve Phenylpropanoid Accumulation in Tomato

**DOI:** 10.3390/molecules27010317

**Published:** 2022-01-05

**Authors:** Xiangyu Ding, Ziyi Yin, Shaoli Wang, Haoqi Liu, Xiaomeng Chu, Jiazong Liu, Haipeng Zhao, Xinyu Wang, Yang Li, Xinhua Ding

**Affiliations:** 1 State Key Laboratory of Crop Biology, Shandong Provincial Key Laboratory of Agricultural Microbiology, College of Plant Protection, Shandong Agricultural University, Tai’an 271018, China; sdauzbdxy@163.com (X.D.); zyyin@sdau.edu.cn (Z.Y.); lhq13573503212@163.com (H.L.); xiaomengchu0721@163.com (X.C.); 2019110147@sdau.edu.cn (J.L.); haipeng@sdau.edu.cn (H.Z.); 17686263885@163.com (X.W.); 2 Yantai Academy of Agricultural Sciences, Yantai 265500, China; shaoliwang123@126.com

**Keywords:** tomato, fruit specific promoters, *AtMYB12*, phenylpropanoid, flavonols, antioxidant capacity

## Abstract

Tomato is an economically crucial vegetable/fruit crop globally. Tomato is rich in nutrition and plays an essential role in a healthy human diet. Phenylpropanoid, a critical compound in tomatoes, reduces common degenerative and chronic diseases risk caused by oxidative stress. As an MYB transcription factor, *ATMYB12* can increase phenylpropanoid content by activating phenylpropanoid synthesis related genes, such as PAL, C4H, 4CL, CHS. However, the heterologous expression of *AtMYB12* in tomatoes can be altered through transgenic technologies, such as unstable expression vectors and promoters with different efficiency. In the current study, the efficiency of other fruit-specific promoters, namely E8S, 2A12, E4, and PG, were compared and screened, and we determined that the expression efficiency of *AtMYB12* was driven by the E8S promoter was the highest. As a result, the expression of phenylpropanoid synthesis related genes was regulated by *AtMYB12*, and the phenylpropanoid accumulation in transgenic tomato fruits increased 16 times. Additionally, the total antioxidant capacity of fruits was measured through Trolox equivalent antioxidant capacity (TEAC) assay, which was increased by 2.4 times in E8S transgenic lines. TEAC was positively correlated with phenylpropanoid content. Since phenylpropanoid plays a crucial role in the human diet, expressing *AtMYB12* with stable and effective fruit-specific promoter E8S could improve tomato’s phenylpropanoid and nutrition content and quality. Our results can provide genetic resources for the subsequent improvement of tomato varieties and quality, which is significant for human health.

## 1. Introduction

Phenylpropanoid is a class of secondary metabolites synthesized from phenylalanine in plants, which mainly includes caffeoylquinic substances and flavonoids, such as caffeoylquinic acids (CQAs), quercetin, quercetin rutinoside (rutin), and kaempferol rutinoside [1,2,3]. Flavonoids play an important role in improving the resilience of plants against various biological and abiotic stresses [4,5]. Rutin, for instance, enhances plant resistance against a variety of bacterial diseases by activating the salicylic acid (SA) synthesis pathway and regulating the expression of disease resistance genes, such as NPR1 [6]. Quercetin could induce H_2_O_2_-mediated pathogen resistance against *Pst* DC3000 in *Arabidopsis thaliana* [7]. Meanwhile, chlorogenic acid, a class of CQAs, has an inhibitory effect on pathogen growth [8,9]. Phenylpropanoid can assist in eliminating ROS and increase drought stress and UV radiation resistance of plants [10,11].

Phenylpropanoid is highly associated with human health due to its anti-oxidant, anti-inflammatory, and anti-viral properties [12,13,14]. Chlorogenic acid can neutralize testicular lesions induced by arsenic because of its oxido-inflammatory stress and apoptotic responses [15,16,17]. Several studies have explored that phenylpropanoid ingestion has diversified effects on human health, primarily cardiovascular disease (CVD), diabetes, cancer, and cognitive disorders [18,19,20,21]. Based on the studies mentioned above, phenylpropanoid is widely used to treat cardiovascular and cerebrovascular diseases and as an anti-tumour drug [22,23,24,25,26].

Phenylpropanoid plays a key role in both plant resistance and human health. Therefore, we must study the synthesis mechanism and regulation of phenylpropanoid. Its biosynthesis is regulated by complex environmental and physiological signals. [2,3]. Two types of genes primarily control phenylpropanoid metabolism: the first type includes structural genes, which directly encode enzymes related to phenylpropane biosynthesis, whereas the others are regulatory genes that control the expression of structural genes. A transcription factor is a protein molecule that regulates multiple genes in the metabolic pathway at the transcription level [27]. MYB transcription factors (TFs) are a large group of regulators, adjusting phenylpropanoid by targeting the promoter region of a key synthase gene in the phenylalanine metabolic pathway, such as *CHS*, *CHI*, *F3H*, *F3′H*. MYB’s *STMTF1* (*MYB1*) in potato (*Solanum tuberosum*) increases flavonols and caffeoylquinic contents by activating the phenylpropanoid pathway [27]. *MdMYB3* in apples can induce flavonols synthesis [28]. Specific expression of *AtMYB12* in tomato fruits increased flavonols and hydroxycinnamates content to as much as 10% of fruit dry weight [29,30]. Moreover, the *AtMYB12* acts as an activating factor for phenylpropanoid synthesis [31].

Transgenic technology effectively improves crop quality by modifying genes controlling sterling phenotypes [32]. At present, transgenic plants are often driven by two promoters to express genes. The first type of promoters includes the constitutive promoter, cauliflower mosaic virus (CaMV) 35S promoter, which can drive the target gene expression in all plant tissue parts and at various developmental stages; 35S promoter is used for constitutive expression of genes, consuming a large amount of energy and nutrients in plants and limiting plant growth and development. The second type of promoters is tissue-specific expression promoters, isolated and expressed in specific tissues. The accurate regulation of gene expression timed quantification will greatly improve the expression efficiency of foreign genes in plants. As an efficient genetic engineering tool, fruit-specific promoters have been used to study the molecular mechanism of fruit development and to improve fruit quality by increasing functional components [33,34,35]. Particularly in tomatoes, fruit ripening-specific promoters are well used and researched. Ethylene responsive promoters [36], such as E8 [37,38,39], E4, PG [40,41], and 2A12 [33,42] are well known and extensively studied tomato fruit-specific promoters. Although these promoters have been used to modify tomatoes genetically, the regulatory efficiency differences and the regulatory model of the phenylpropanoid synthesis pathway are still unclear. E4 and E8 exhibited a higher expression level in fruits than in the leaves, and their expression levels increased significantly as the fruit ripening proceeded [30,43,44,45]. PG initiator was cloned from the multi-polymer semi-lactate PG gene, found only in ripe tomato fruits [46]. Among the fruit-specific promoters, 2A11 is one of the highly specific promoters, and the 2A12 promoter was amplified by PCR based on the 2A11 promoter and had strict fruit expression specificity [42,47,48].

Tomato is a popular vegetable/fruit globally because it contains abundant nutrients, such as phenylpropanoid, lycopene, and vitamins. Among these, phenylpropanoid, the primary source of dietary polyphenols, are bioavailable molecules in humans with impressive health benefits, such as antioxidation, cardioprotection, antibacterial, anti-viral, and anticancer activity [49]. Phenylpropanoid metabolism in tomatoes is well established and reforming an efficient strategy to enhance phenylpropanoid in tomatoes is crucial [50]. Since phenylpropanoid biosynthesis is dominated by a series of genes, the use of transcription factors activates or suppresses metabolism-related genes, providing effective ways to engineer plants enriched with valuable secondary metabolites [2]. Although studies revealed that, as an MYB transcription factor, *AtMYB12* allows positive regulation of flavonoid biosynthesis, the stability and effectiveness of the *AtMYB12* expression in fruit varies widely among proponents [30,51]. In the current study, *AtMYB12* was driven by four fruit-specific promoters, downstream regulatory gene expression, and phenylpropanoid accumulation. The antioxidant activity in fruits was analysed uniformly. The high-efficiency expression of genes related to phenylpropanoid synthesis driven by E8S promoter in fruits can improve the favourable metabolites content of tomatoes effectively and reduce the energy and nutrient consumption caused by constitutive promoters, which will not limit the growth and development of plants [52]. Meanwhile, the antioxidant capacity of tomatoes increased significantly with the increasing phenylpropanoid content. Adding tomatoes to daily diets can improve antioxidant capacity, scavenge free radicals, and delay ageing. The current study provides a basis for cultivating high phenylpropanoid content in tomato products, which are of great importance to human health.

## 2. Results

### 2.1. Effects of Fruit-Specific Promoters Regulating AtMYB12 Expression in Tomato Fruits

**Figure 1:**
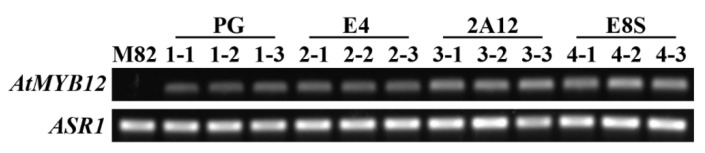
RT-PCR analysis of *AtMYB12* expression in wild type and transgenic lines with different promoters. M82: Wild type; 1-1: PG-1-1, 1-2: PG-1-2, 1-3: PG-1-3;2-1: E4-2-1, 2-2: E4-2-2, 2-3: E4-2-3; 3-1: 2A12-3-1, 3-2: 2A12-3-2, 3-3: 2A12-3-3; 4-1: E8S-4-1, 4-2: E8S-4-2, 4-3: E8S-4-3; ASR1(LOC543574) was used as a control.

To obtain *AtMYB12* transgenic lines under different fruit-specific promoters, PG, E4, 2A12, and E8S sequences were cloned from tomato *Solanum lycopersicum* ver. Zhongshu No.4 and the *AtMYB12* was cloned from *Arabidopsis thaliana* col-0, respectively. Three lines were selected from each promoter transgenic plant of red maturity (E4-1-1, E4-1-2, E4-1-3, PG-2-1, PG-2-2, PG-2-3, 2A12-3-1, 2A12-3-2, 2A12-3-3, E8S-4-1, E8S-4-2, E8S-4-3) and the exocarp peel for further tests. Results revealed that different fruit-specific promoters could successfully drive *AtMYB12* in tomatoes with different efficiency. E8S and 2A12 promoters exhibited a relatively higher expression level, whereas E4 and PG expressions were relatively low. These results indicated that the efficiency of *AtMYB12* expression varies dramatically with different fruit-specific promoters (Figure 1).

### 2.2. Expression of AtMYB12 Driven by Different Fruit Specific Promoters Increased Phenylpropanoid Accumulation in Varying Degrees

To evaluate the content of phenylpropanoid in T_0_-generation strains, HPLC assay was carried out and discovered that the total amount of several major phenylpropanoids could reach up to 15.41 mg/g in the E8S transgenic lines, which is 15.89-fold of wild type tomatoes, followed with 2A12, E4, and PG transgenic lines with the highest total content of phenylpropanoid as high as 5.88-fold, 4.62-fold, and 4.29-fold of wild tomatoes, respectively (Table 1, Table 2, Table 3 and Table 4).

**Figure 2:**
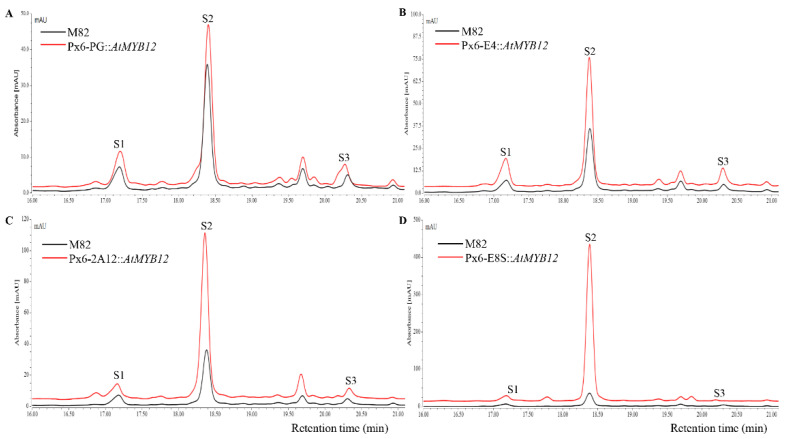
HPLC analysis of extracts from wild-type and *AtMYB12*-expressing tomatoes. (**A**) Comparison of HPLC profiles between wild type and pX6-PG::*AtMYB12*-expressing tomatoes; (**B**) Comparison of HPLC profiles between wild type and pX6-E4::*AtMYB12*-expressing tomatoes; (**C**) Comparison of HPLC profiles between wild type and pX6-2A12::*AtMYB12*-expressing tomatoes; (**D**) Comparison of HPLC profiles between wild type and pX6-E8S::*AtMYB12*-expressing tomatoes. S1: dicaffeoylquinic acid; S2: quercetin rutinoside (rutin); S3: kaempferol rutinoside.

Phenylpropanoids were extracted from mature T_1_ transgenic tomato peels, and three kinds of representative essential phenylpropanoids were detected using high-efficiency liquid chromatography technology (HPLC), including quercetin rutinoside (rutin), dicaffeoylquinic acid, and kaempferol rutinoside (Figure 2). Liquid chromatography revealed that compared with the wild type, the phenylpropanoid content of the transgenic lines were increased to varying degrees. Among the transgenic lines, px6-E8S::*AtMYB12* transgenic fruits exhibited the highest peak and area, indicating the highest phenylpropanoid content, followed by px6-2A12::*AtMYB12*, px6-E4::*AtMYB12*, and px6-PG::*AtMYB12* transgenic lines.

The content of phenylpropanoid in the T_1_ generation was also tested, and similar results were obtained, revealing that phenylpropanoid synthesis efficiency sorted from high to low were as follows: E8S, 2A12, E4, and PG, with a maximum content of 12.53 mg/g, 4.89 mg/g, 4.15 mg/g, and 3.24 mg/g, which are 14.91-fold, 5.82-fold, 4.94-fold, 3.86-fold higher than the wild type, respectively (Table 5). To sum up, the ability of *AtMYB12* driven by different promoters to facilitate phenylpropanoid synthesis was E8S, 2A12, followed by E4 and PG promoter.

### 2.3. Expression of AtMYB12 Alters the Genes Expressed in the Phenylpropanoid Biosynthesis Pathway

**Figure 3:**
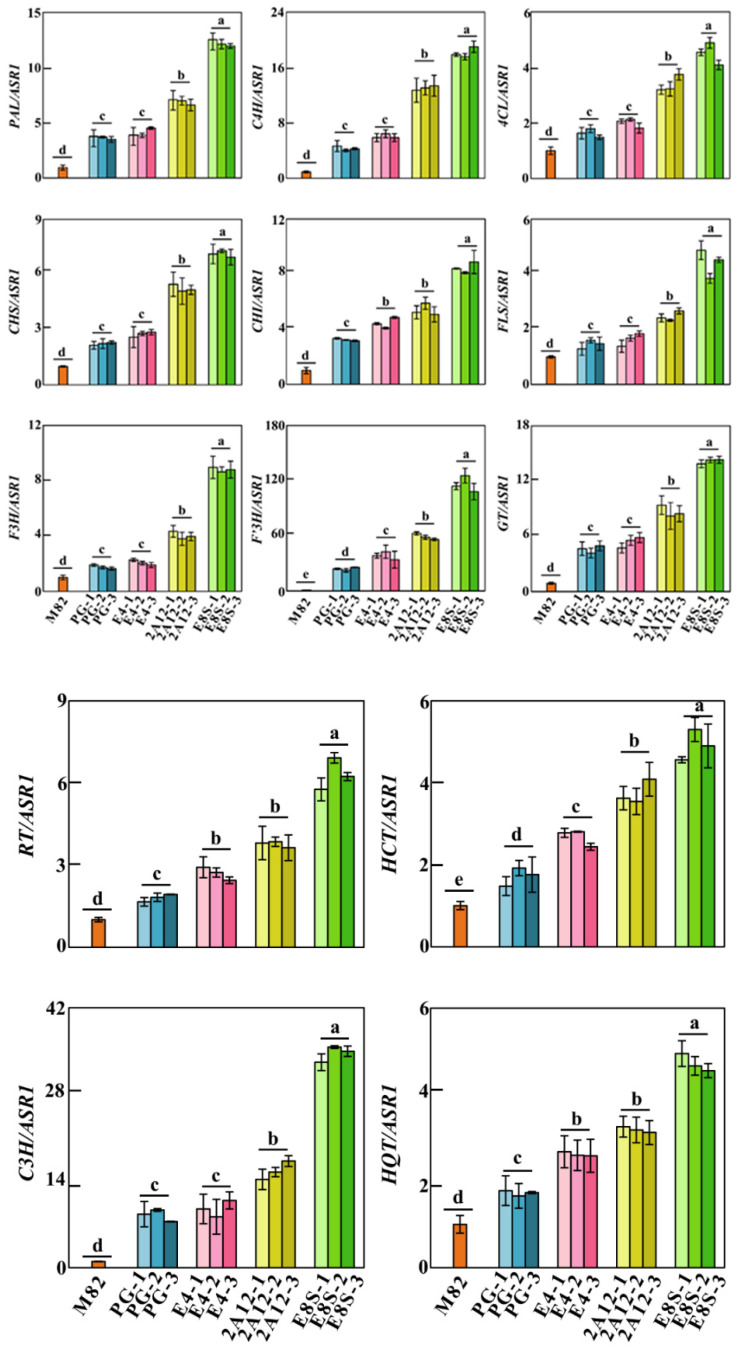
Analysis of transcriptional level of biosynthesis related genes of phenylpropanes by qRT-PCR. Values were normalized to actin expression. Error bars represent SD of three independent biological replicates. PAL: phenylalanine ammonia lyase, C4H: cinnamate 4-hydroxylase, 4CL: 4-hydroxycinnamoyl CoA ligase, CHS: chalcone synthase, CHI: chalcone isomerase, FLS: flavonol synthase, F3H: flavanone-3-hydroxylase, F3′H: flavonoid-3′-hydroxylase, GT: flavonol-3- glucosyltransferase, RT: flavonol-3-glucoside-rhamnosyltransferase, C3H: p-coumaroyl ester 3-hydroxylase, HCT: hydroxycinnamoyl CoA shikimate/quinate transferase and HQT: hydroxycinnamoyl CoA quinate transferase. Different letters indicate samples with statistical differences, Student’s *t*-test; *n* = 3.

Since the significant change of phenylpropanoid content is related in different promoter-driven transgenic plants, we hypothesized if the result was due to gene changes linked to the phenylpropanoid biosynthesis pathway. Thus, the expression levels of phenylpropanoid pathway genes [2], including phenylalanine ammonia-lyase (*PAL*), cinnamate 4-hydroxylase (*C4H*), 4-hydroxycinnamoyl CoA ligase (*4CL*), chalcone synthase (*CHS*), chalcone isomerase (*CH*)*I*, flavonol synthase (*FLS*), flavanone-3-hydroxylase (*F3H*), flavonoid-3′-hydroxylase (*F3′H*), flavonol-3-glucosyltransferase (*GT*), flavonol-3-glucoside-rhamnosyltransferase (*RT*), flavanone-3-hydroxylase (*C3H*), hydroxycinnamoyl CoA shikimate/quinate transferase (*HCT*), and hydroxycinnamoyl CoA quinate transferase (*HQT*) were examined through qRT-PCR in T_1_ generation fruits and were compared with the control. In Px6-E8S::*AtMYB12* transgenic fruits, we observed a 128.71-fold increase in *F3′H*, 13.02-19.65-fold increase in *PAL*, *C4H*, and *GT*, 4.414 to 8.81-fold increase in *FLS*, *4CL*, *RT*, *CHS*, *CHI*, and *F3H* relative to the levels in wild-type tomato peel. In Px6-2A12::*AtMYB12* transgenic fruits, a 14.13–65.63-fold increase in *C4H* and *F3′H*, and a 2.47–9.65-fold increase in other genes related to flavonol synthesis. In Px6-E4::*AtMYB12* and Px6-PG::*AtMYB12* transgenic fruits, all the synthetic genes were upregulated to varying degrees, and the variation was relatively low compared with the previous two transgenic lines. In addition to flavonol synthesis, genes that are essential for CGA synthesis, namely *C3H*, *HQT*, and *HCT*, were upregulated by *AtMYB12* expression (Figure 3). These results revealed that fruit-specific promoters expression of *AtMYB12* in tomatoes led to biosynthetic genes induction required for phenylpropanoid production.

### 2.4. Expressing of AtMYB12 with Fruit Specific Promoters Lead to a Significant Rise of Total Antioxidant Capacity

**Figure 4:**
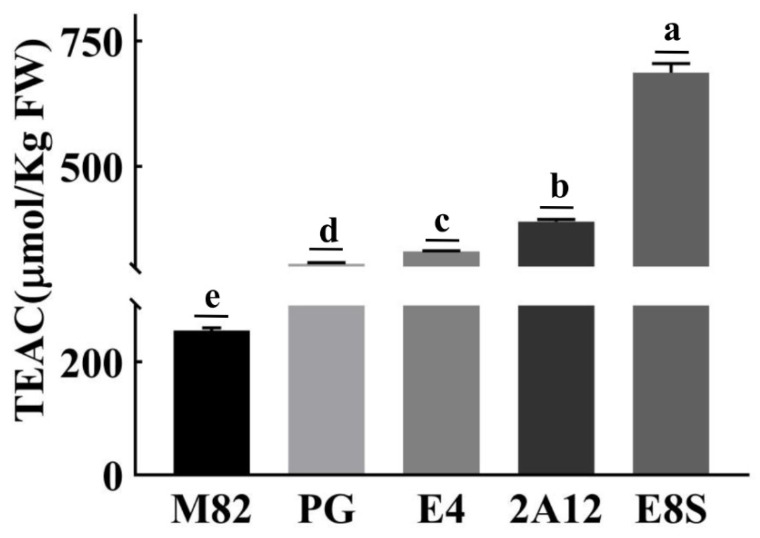
Total antioxidant capacity in wild-type (M82) and T_1_ generation *AtMYB12*-expressing tomato fruits. Fresh tomato peel antioxidant activities in mature tomato fruits of each cultivar. Tree different tomato fruits of each cultivar were pooled for detection. Different letters indicate samples with statistical differences, Student’s *t*-test; *n* = 3.

Total antioxidant capacity was measured using TEAC assay. In *AtMYB12* transgenic fruits, the TEAC activity of the water-soluble fraction (containing phenolics) is significantly increased up to 2.4-fold compared with the control (Figure 4). The antioxidant ability of different genetically modified strains exhibited the strongest antioxidant capacity of E8S (687.04 µm/Kg) fresh weight, and the weakest of PG (305.96 µm/Kg FW), which is positively related to the content of phenylpropanoid.

## 3. Discussion

As the crucial secondary metabolites, phenylpropanoid plays an important role in human health. Its biosynthesis is dominated by a highly complex process intimately related to regulating various genes and transcription factors, such as *AtMYB12* in plants [2,3]. Overexpression of genes related to synthesis regulation by transgenic technology is an effective method to increase the content of phenylpropanoid in plants [27,32,53]. However, it is still limited by promoter selection and gene expression efficiency. CaMV 35S promoter is widely considered a strong constitutive promoter. However, this promoter does not confer any specificity: neither tissue-specificity nor plant developmental stage-specificity on exogenous gene expression leading to lower expression levels [37]. In the current case, the tissue-specific promoter is an excellent alternative, which could accurately regulate gene expressions timely and quantitatively and greatly improve foreign gene expression efficiency. In the current study, by comparing the efficiency of different fruit-specific promoters of PG, E4, 2A12, and E8S promoter, we discovered that the stable and effective fruit-specific promoter E8S could dramatically increase the gene expression of *AtMYB12*, which in turn promoted the accumulation of phenylpropanoid and improved the antioxidant capacity of tomato fruit. 

Moreover, the contents of phenylpropanoid in the genetically modified plants with different fruit-specific promoters were promoted, suggesting a direct link between *AtMYB12* gene expression and phenylpropanoid content. QRT-PCR was carried out to analyse the expression efficiency of other essential enzyme genes during the synthesis of different substances in the phenylpropane pathway [3,54]. The synthase related genes were significantly increased, which was correlated with phenylpropanoid content.

**Figure 5:**
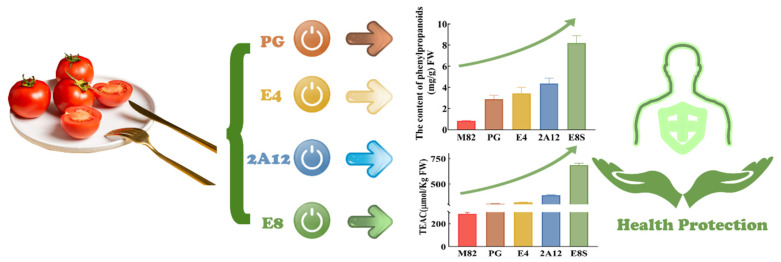
Model of different promoters driven AtMYB12 expression leads to improvements of phenylpropanoid accumulation in tomato.

The −2181 to −1088 region of E8 is the crucial regulatory element essential for its response to ethylene [55]. E8S in our research is located in this region, and the most excellent tomato fruit specific promoter we selected was E8S, which significantly positively regulates *AtMYB12* expression. Those results are consistent with the previous study. The genes related to the phenylpropanoid synthesis pathway regulated by *AtMYB12* were up-regulated considerably, and the phenylpropanoid content increased. The phenylpropanoid content was significantly positively correlated with the antioxidant capacity of phenolic compounds, including phenolic acids, flavonoids and proanthocyanidins, which are widely distributed in plants as a protective mechanism against biotic, abiotic stresses. Fruits, vegetables, grains, spices, and herbs are the richest source of dietary phenylpropanoid. High intake of these foods like tomatoes has been linked to lowered risk of most common degenerative and chronic diseases known to be caused by oxidative stress [56,57]. In this study, by comparing the efficiency of different fruit specific promoters, the best one was selected for genetic improvement to increase phenylpropanoid content and improve fruit quality. Simultaneously, the accumulation of phenylpropanoid increases the total antioxidant capacity of tomatoes, slows down the accumulation of reactive oxygen species, thus delaying the overripening of tomatoes [58]. In general, since phenylpropanoids are tightly relevant to human health, our findings provided genetic resources for the subsequent improvement of tomato varieties and quality (Figure 5). Additionally, reports on whether phenylpropanoids can delay senescence in tomato fruit storage or not are pretty few and exploring its effect on postharvest storage would be beneficial. These studies are currently underway.

## 4. Materials and Methods

### 4.1. Plant Materials and Growth Conditions

Plants of M82 (*Solanum lycopersicum* cv. M82) and the transgenic lines with different promoters, were grown in a greenhouse under a 16 h light/8 h dark cycle at 25 °C, with 70% relative humidity. Ten days after fruit breaker stage, sample was taken for study.

### 4.2. Strain Construction and Transformation

The E8S (GenBank: KJ561284), E4 (GenBank: S44898), 2A12 (GenBank: X07410), PG (GenBank: DQ453963) promoters were amplified from *Solanum lycopersicum* ver. Zhongshu No.4. The full-length cDNA of *AtMYB12* (AT2G47460) was amplified from *Arabidopsis thaliana* (ecotype: Columbia) (Table S1). The DNA of the PG, E4, 2A12, E8S promoter were digested with *Xho*I and *Spe*I and then ligated into *Xho*I/*Spe*I digested pX6, replacing GFP, to produce the transitional vector pX6-promoter. The digested full-length *AtMYB12* CDS was inserted into pX6-promoter to produce the transitional vector pX6-PG::*AtMYB12*, pX6-E4::*AtMYB12*, pX6-2A12::*AtMYB12*, pX6-E8S::*AtMYB12*. This construct was transformed into *Agrobacterium tumefaciens* strain AGL1 by electroporation. *Agrobacterium*-mediated transformation into tomato cotyledon explants was performed using a previously published method [59,60].

### 4.3. Quantifcation of Phenylpropanoid

The major phenylpropanoids were extracted from the exocarp of tomato and the 0.2 g skin samples were ground to powder in liquid nitrogen and extracted overnight at −20 °C in 2 mL methanol with 100% chromatographic grade purity (Sigma-Aldrich, Saint Louis, MO, USA). The first 2 h were shaken and mixed every 15 min for full extraction. The sample was centrifuged at 4 °C and 4000 g for 20 min. The supernatant was filtered with 0.22 µm microporous membrane and stored in dark at −20 °C. The phenylpropanoids were quantified by HPLC (high-performance liquid chromatography, Agilent Technologies 1200 series, Beijing, China) with a chromatographic column (Agilent Technologies ZORBAX SB-C18 4.6 × 250 mm). Aqueous phase A was 0.1% acetic acid solution; organic phase B was pure methanol. The gradient as follow: t = 0.0 min, 20% B; t = 10 min, 30% B; t = 25 min, 90% B; t = 27 min, 90% B; t =28 min, 20% B; t = 32 min, 20% B, each sample was injected 10 µL and the flow rate was 1 mL/min. Detection by ultraviolet (UV) chromatograms was recorded at 325 nm and the column temperature was 35 °C [30]. All phenylpropanoid standards, rutin, naringenin chalcone and kaempferol rutinoside were obtained from Sigma-Aldrich.

### 4.4. Quantitative Reverse-Transcription PCR(qRT-PCR)

The concentration and purity of the RNA samples were determined by UV absorbance spectrophotometry (260 nm/280 nm ratio). First-strand cDNA was synthesized using Super Quick RT Master Mix (CWBIO, Beijing, China) following the manufacturer’s instructions. Transcription of phenylpropanoid biosynthetic genes were analyzed by qRT-PCR using gene-specific primers (Table S1). All target gene confirmations were performed using SYBR Premix Ex Taq (Takara, Dalian, China). All tomato quantifications were normalized to the abscisic stress ripening gene 1 (GenBank: L08255.1); these genes were amplified under the same conditions. QRT-PCR was conducted on the Bio-Rad iQTM5 Light Cycler analysis system with SYBR^®^ Premix Ex Taq^TM^ (Tli RNaseH Plus). The ASR1 gene was used as an internal control to standardize the results. We mixed plant tissues from all three T_1_ progeny together to detect the expression of phenylpropanoid biosynthetic genes between the varieties of tomato and the different transgenic tomato lines. All experiments were carried out with three biological repeats and four technical trials.

### 4.5. Total Antioxidant Activity

The extraction method of phenylpropanoid was consistent with the description in 4.3. To measure antioxidant capacity, we performed the 2,2′-azinobis (3-ethylbenzothiazoline-6-sulfonic acid) (ABTS). Trolox equivalent antioxidant capacity (TEAC) assay, which measures the ability of compounds to scavenge the ABTS radical cation (ABTS+) in relation to Trolox (6-hydroxy-2,3,7,8-tetramethylchroman-2-carboxylic acid; Sigma). The results were expressed as the TEAC in µmol of trolox per kg of dry weight. All experiments were carried out with three biological repeats and three technical trials.

### 4.6. Statistical Analyses

Each value represents repeated independent experiments, and the vertical bars expressed the arithmetic means ± standard deviations (SD). Tukey’s test was used to calculate statistical significance, and the significant differences between treatments and the untreated control are represented by a, b, c.

## 5. Conclusions

In conclusion, E8S is the most efficient promoter. *AtMYB12* driven by E8S promoter positive regulation of the phenylpropanoid accumulation in transgenic tomato fruits. In the meanwhile, the antioxidant capacity of tomato was enormously improved which was positively correlated with phenylpropanoid content. Since phenylpropanoids play an important role in the human diet, our results of expressing *AtMYB12* with stable and effective fruit specific promoter E8S providing genetic resources for the subsequent improvement of tomato nutrition and quality. Adding tomatoes that rich in phenylpropanoid to the diet has great significance for human health.

## Figures and Tables

**Table 1:** Quantification of major phenylpropanoids in T_0_ wild-type (M82) and px6-PG::*AtMYB12*-expressing tomatoes.

Line	diCQA ^1^ (mg/g) FW ^4^	Fold Increase	QueRut ^2^ (mg/g) FW	Fold Increase	KaeRut ^3^ (mg/g) FW	Fold Increase	Phenylpro-Panoids (mg/g) FW	Fold Increase
M82 ^5^	0.23 ± 0.05 ^a^		0.59 ± 0.07 ^a^		0.15 ± 0.02 ^a^		0.97 ± 0.11 ^a^	
PPGA ^6^-1	0.46 ± 0.09 ^b^	2.00	1.25 ± 0.15 ^b^	2.12	1.02 ± 0.16 ^b^	6.80	2.73 ± 0.19 ^b^	2.81
PPGA-2	0.85 ± 0.17 ^b^	3.70	2.01 ± 0.17 ^b^	3.41	0.90 ± 0.10 ^b^	6.00	3.76 ± 0.23 ^b^	3.88
PPGA-3	1.02 ± 0.11 ^b^	4.43	2.11 ± 0.13 ^b^	3.58	1.03 ± 0.09 ^b^	6.87	4.16 ± 0.17 ^b^	4.29
PPGA-4	0.94 ± 0.02 ^b^	4.09	1.74 ± 0.10 ^b^	2.95	0.81 ± 0.12 ^b^	5.40	3.49 ± 0.20 ^b^	3.60
PPGA-5	0.76 ± 0.09 ^b^	3.30	1.67 ± 0.09 ^b^	2.83	0.92 ± 0.15 ^b^	6.13	3.35 ± 0.15 ^b^	3.45
PPGA-6	0.67 ± 0.04 ^b^	2.91	1.36 ± 0.04 ^b^	2.31	1.04 ± 0.09 ^b^	6.93	3.07 ± 0.09 ^b^	3.16
PPGA-7	0.88 ± 0.07 ^b^	3.83	1.50 ± 0.07 ^b^	2.54	1.02 ± 0.10 ^b^	6.80	3.40 ± 0.15 ^b^	3.51
PPGA-8	0.64 ± 0.05 ^b^	2.78	1.45 ± 0.05 ^b^	2.46	0.95 ± 0.05 ^b^	6.33	3.04 ± 0.09 ^b^	3.13
PPGA-9	0.71 ± 0.10 ^b^	3.09	1.40 ± 0.05 ^b^	2.37	0.72 ± 0.09 ^b^	4.80	2.83 ± 0.11 ^b^	2.92
PPGA-10	0.82 ± 0.04 ^b^	3.57	1.55 ± 0.09 ^b^	2.63	0.74 ± 0.12 ^b^	4.93	3.11 ± 0.18 ^b^	3.21

^1^ diCQA, dicaffeoylquinic acid; ^2^ QueRut, quercetin rutinoside (rutin); ^3^ KaeRut, kaempferol rutinoside; ^4^ FW, fresh weight; ^5^ M82, wild type; ^6^ PGA, Px6-PG::*AtMYB12*-expressing tomatoes. Note: Each value represents the average (±SD) of 3 fruits of different lines. SD are standard deviation. Different letters in the same column indicate significant differences at the level of 0.05.

**Table 2:** Quantification of major phenylpropanoids in T_0_ wild-type (M82) and px6-E4::*AtMYB12*-expressing tomatoes.

Line	diCQA ^1^ (mg/g) FW ^4^	Fold Increase	QueRut ^2^ (mg/g) FW	Fold Increase	KaeRut ^3^ (mg/g) FW	Fold Increase	Phenylpro-Panoids (mg/g) FW	Fold Increase
M82 ^5^	0.23 ± 0.04 ^a^		0.59 ± 0.01 ^a^		0.15 ± 0.02 ^a^		0.97 ± 0.06 ^a^	
PE4A ^6^-1	1.45 ± 0.07 ^b^	6.30	2.35 ± 0.28 ^b^	3.98	0.68 ± 0.14 ^b^	4.53	4.48 ± 0.11 ^b^	4.62
PE4A-2	1.35 ± 0.15 ^b^	5.87	2.02 ± 0.15 ^b^	3.42	0.73 ± 0.07 ^b^	4.87	4.11 ± 0.09 ^b^	4.24
PE4A-3	1.31 ± 0.18 ^b^	5.70	2.01 ± 0.33 ^b^	3.41	0.55 ± 0.08 ^b^	3.67	3.87 ± 0.04 ^b^	3.99
PE4A-4	1.01 ± 0.08 ^b^	4.39	1.88 ± 0.42 ^b^	3.19	0.21 ± 0.01 ^b^	1.40	3.10 ± 0.02 ^b^	3.20
PE4A-5	0.67 ± 0.02 ^b^	2.91	1.73 ± 0.10 ^b^	2.93	0.20 ± 0.09 ^b^	1.33	2.60 ± 0.04 ^b^	2.68
PE4A-6	0.81 ± 0.07 ^b^	3.52	2.02 ± 0.01 ^b^	3.42	0.44 ± 0.07 ^b^	2.93	3.27 ± 0.10 ^b^	3.37
PE4A-7	0.59 ± 0.11 ^b^	2.57	1.99 ± 0.84 ^b^	3.37	0.36 ± 0.17 ^b^	2.40	2.93 ± 0.09 ^b^	3.02
PE4A-8	0.47 ± 0.08 ^b^	2.04	2.46 ± 0.66 ^b^	4.17	0.52 ± 0.02 ^b^	3.47	3.46 ± 0.15 ^b^	3.57
PE4A-9	0.62 ± 0.11 ^b^	2.70	2.05 ± 0.07 ^b^	3.47	0.35 ± 0.14 ^b^	2.33	3.02 ± 0.04 ^b^	3.11
PE4A-10	0.77 ± 0.02 ^b^	3.35	1.93 ± 0.10 ^b^	3.27	0.74 ± 0.04 ^b^	4.93	3.45 ± 0.07 ^b^	3.56

^1^ diCQA, dicaffeoylquinic acid; ^2^ QueRut, quercetin rutinoside (rutin); ^3^ KaeRut, kaempferol rutinoside; ^4^ FW, fresh weight; ^5^ M82, wild type; ^6^ PE4A, Px6-E4::*AtMYB12*-expressing tomatoes. Different letters in the same column indicate significant differences at the level of 0.05.

**Table 3:** Quantification of major phenylpropanoids in T_0_ wild-type (M82) and px6-2A12::*AtMYB12*-expressing tomatoes.

Line	diCQA ^1^ (mg/g) FW ^4^	Fold Increase	QueRut ^2^ (mg/g) FW	Fold Increase	KaeRut ^3^ (mg/g) FW	Fold Increase	Phenylpro-Panoids (mg/g) FW	Fold Increase
M82 ^5^	0.23 ± 0.01 ^a^		0.59 ± 0.07 ^a^		0.15 ± 0.01 ^a^		0.97 ± 0.03 ^a^	
P2AA ^6^-1	0.56 ± 0.07 ^b^	2.43	3.51 ± 0.21 ^b^	5.95	1.03 ± 0.06 ^b^	6.87	5.10 ± 0.21 ^b^	5.26
P2AA-2	0.65 ± 0.03 ^b^	2.83	3.45 ± 0.15 ^b^	5.85	0.89 ± 0.04 ^b^	5.93	5.00 ± 0.19 ^b^	5.15
P2AA-3	0.32 ± 0.04 ^b^	1.39	3.13 ± 0.10 ^b^	5.31	1.22 ± 0.08 ^b^	8.13	4.68 ± 0.11 ^b^	4.82
P2AA-4	0.79 ± 0.06 ^b^	3.43	2.85 ± 0.09 ^b^	4.83	0.95 ± 0.04 ^b^	6.33	4.58 ± 0.07 ^b^	4.72
P2AA-5	1.21 ± 0.10 ^b^	5.26	2.68 ± 0.07 ^b^	4.54	1.12 ± 0.09 ^b^	7.47	5.02 ± 0.13 ^b^	5.18
P2AA-6	1.03 ± 0.06 ^b^	4.48	1.55 ± 0.06 ^b^	2.63	0.54 ± 0.02 ^b^	3.60	3.13 ± 0.04 ^b^	3.23
P2AA-7	0.95 ± 0.01 ^b^	4.13	3.01 ± 0.17 ^b^	5.10	1.73 ± 0.13 ^b^	11.53	5.70 ± 0.23 ^b^	5.88
P2AA-8	1.12 ± 0.04 ^b^	4.87	2.02 ± 0.11 ^b^	3.42	1.25 ± 0.09 ^b^	8.33	4.40 ± 0.15 ^b^	4.54
P2AA-9	0.89 ± 0.02 ^b^	3.87	2.01 ± 0.04 ^b^	3.41	0.71 ± 0.02 ^b^	4.73	3.60 ± 0.09 ^b^	3.71
P2AA-10	1.02 ± 0.05 ^b^	4.43	2.42 ± 0.11 ^b^	4.10	1.01 ± 0.06 ^b^	6.73	4.44 ± 0.20 ^b^	4.58

^1^ diCQA, dicaffeoylquinic acid; ^2^ QueRut, quercetin rutinoside (rutin); ^3^ KaeRut, kaempferol rutinoside; ^4^ FW, fresh weight; ^5^ M82, wild type; ^6^ P2AA, Px6-2A12::*AtMYB12*-expressing tomatoes. Different letters in the same column indicate significant differences at the level of 0.05.

**Table 4:** Quantification of major phenylpropanoids in T_0_ wild-type (M82) and px6-E8S::*AtMYB12*-expressing tomatoes.

Line	diCQA ^1^ (mg/g) FW ^4^	Fold Increase	QueRut ^2^ (mg/g) FW	Fold Increase	KaeRut ^3^ (mg/g) FW	Fold Increase	Phenylpro-Panoids (mg/g) FW	Fold Increase
M82 ^5^	0.23 ± 0.02 ^a^		0.59 ± 0.02 ^a^		0.15 ± 0.01 ^a^		0.97 ± 0.05 ^a^	
PE8SA ^6^-1	2.84 ± 0.09 ^b^	12.35	10.25 ± 0.41 ^b^	17.37	2.32 ± 0.10 ^b^	15.47	15.41 ± 0.26 ^b^	15.89
PE8SA-2	2.15 ± 0.04 ^b^	9.35	5.12 ± 0.26 ^b^	8.68	2.00 ± 0.07 ^b^	13.33	9.28 ± 0.21 ^b^	9.57
PE8SA-3	1.32 ± 0.01 ^b^	5.74	3.15 ± 0.10 ^b^	5.34	1.01 ± 0.08 ^b^	6.73	5.49 ± 0.14 ^b^	5.66
PE8SA-4	0.56 ± 0.02 ^b^	2.43	1.12 ± 0.07 ^b^	1.90	3.01 ± 0.11 ^b^	20.07	4.70 ± 0.11 ^b^	4.85
PE8SA-5	1.22 ± 0.03 ^b^	5.30	6.12 ± 0.21 ^b^	10.37	2.14 ± 0.09 ^b^	14.27	9.48 ± 0.17 ^b^	9.77
PE8SA-6	1.95 ± 0.09 ^b^	8.48	2.15 ± 0.10 ^b^	3.64	0.90 ± 0.01 ^b^	6.00	5.01 ± 0.10 ^b^	5.16
PE8SA-7	1.02 ± 0.03 ^b^	4.43	5.15 ± 0.25 ^b^	8.73	0.55 ± 0.06 ^b^	3.67	6.72 ± 0.09 ^b^	6.93
PE8SA-8	0.49 ± 0.08 ^b^	2.13	9.23 ± 0.33 ^b^	15.64	1.25 ± 0.09 ^b^	8.33	10.96 ± 0.21 ^b^	11.30
PE8SA-9	0.98 ± 0.04 ^b^	4.26	4.22 ± 0.17 ^b^	7.15	0.85 ± 0.07 ^b^	5.67	6.05 ± 0.15 ^b^	6.24
PE8SA-10	0.87 ± 0.01 ^b^	3.78	3.92 ± 0.11 ^b^	6.64	0.68 ± 0.02 ^b^	4.53	5.48 ± 0.18 ^b^	5.65

^1^ diCQA, dicaffeoylquinic acid; ^2^ QueRut, quercetin rutinoside (rutin); ^3^ KaeRut, kaempferol rutinoside; ^4^ FW, fresh weight; ^5^ M82, wild type; ^6^ PE8SA, Px6-E8S::*AtMYB12*-expressing tomatoes. Different letters in the same column indicate significant differences at the level of 0.05.

**Table 5:** Quantification of major phenylpropanoids in T_1_ wild-type (M82) and px6-promoters::*AtMYB12*-expressing tomatoes.

Line	diCQA ^1^ (mg/g) FW ^4^	Fold Increase	QueRut ^2^ (mg/g) FW	Fold Increase	KaeRut ^3^ (mg/g) FW	Fold Increase	Phenylpro-Panoids (mg/g) FW	Fold Increase
M82 ^5^	0.21 ± 0.02 ^a^		0.51 ± 0.05 ^a^		0.12 ± 0.01 ^a^		0.84 ± 0.02 ^a^	
PPGA ^6^-1	0.43 ± 0.06 ^b^	2.05	1.29 ± 0.07 ^b^	2.53	0.76 ± 0.01 ^b^	6.33	2.48 ± 0.10 ^b^	2.95
PPGA-4	0.93 ± 0.06 ^b^	4.43	1.69 ± 0.07 ^b^	3.31	0.62 ± 0.01 ^b^	5.17	3.24 ± 0.10 ^b^	3.86
PPGA-5	0.75 ± 0.09 ^b^	3.57	1.71 ± 0.09 ^b^	3.35	0.65 ± 0.06 ^b^	5.42	3.11 ± 0.09 ^b^	3.70
PPGA-10	0.50 ± 0.06 ^b^	2.67	1.71 ± 0.09 ^b^	3.35	0.52 ± 0.03 ^b^	4.33	2.73 ± 0.08 ^b^	3.25
PE4 ^7^A-1	1.40 ± 0.05 ^b^	6.67	2.20 ± 0.16 ^b^	4.31	0.55 ± 0.03 ^b^	4.58	4.15 ± 0.14 ^b^	4.94
PE4A-3	1.24 ± 0.01 ^b^	5.90	1.85 ± 0.11 ^b^	3.63	0.50 ± 0.02 ^b^	4.17	3.59 ± 0.10 ^b^	4.27
PE4A-4	1.04 ± 0.04 ^b^	4.95	1.64 ± 0.03 ^b^	3.22	0.23 ± 0.01 ^b^	1.92	2.91 ± 0.03 ^b^	3.46
PE4A-6	0.83 ± 0.09 ^b^	3.95	1.88 ± 0.03 ^b^	3.69	0.37 ± 0.03 ^b^	3.08	3.08 ± 0.11 ^b^	3.67
P2AA ^8^-2	0.65 ± 0.03 ^b^	3.10	2.76 ± 0.33 ^b^	5.41	0.90 ± 0.01 ^b^	7.50	4.31 ± 0.10 ^b^	5.13
P2AA-5	1.08 ± 0.19 ^b^	5.14	2.63 ± 0.11 ^b^	5.16	0.82 ± 0.03 ^b^	6.83	4.53 ± 0.10 ^b^	5.39
P2AA-7	0.92 ± 0.03 ^b^	4.38	2.68 ± 0.03 ^b^	5.25	1.29 ± 0.01 ^b^	10.75	4.89 ± 0.03 ^b^	5.82
P2AA-9	0.69 ± 0.05 ^b^	3.29	2.34 ± 0.05 ^b^	4.59	0.67 ± 0.01 ^b^	5.58	3.70 ± 0.07 ^b^	4.40
PE8SA ^9^-1	2.54 ± 0.03 ^b^	12.10	8.27 ± 0.14 ^b^	16.22	1.72 ± 0.08 ^b^	14.33	12.53 ± 0.19 ^b^	14.91
PE8SA-2	2.08 ± 0.20 ^b^	9.90	5.81 ± 0.17 ^b^	11.39	0.99 ± 0.01 ^b^	8.25	8.88 ± 0.20 ^b^	10.57
PE8SA-6	1.65 ± 0.04 ^b^	7.85	4.01 ± 0.01 ^b^	7.86	0.77 ± 0.02 ^b^	6.42	6.43 ± 0.25 ^b^	7.65
PE8SA-8	0.57 ± 0.17 ^b^	4.75	5.48 ± 0.44 ^b^	10.75	0.94 ± 0.01 ^b^	7.83	6.99 ± 0.21 ^b^	8.32

^1^ diCQA, dicaffeoylquinic acid; ^2^ QueRut, quercetin rutinoside (rutin); ^3^ KaeRut, kaempferol rutinoside; ^4^ FW, fresh weight; ^5^ M82, wild type; ^6^ PPGA, Px6-PG::*AtMYB12*-expressing tomatoes; ^7^ PE4A, Px6-E4::*AtMYB12*-expressing tomatoes; ^8^ P2AA, Px6-2A12::*AtMYB12*-expressing tomatoes; ^9^ PE8SA, Px6-E8S::*AtMYB12*-expressing tomatoes. Different letters in the same column indicate significant differences at the level of 0.05.

## Data Availability

All data used to support the findings of this study are included within the article and they are also available from the corresponding author upon request.

## Supplementary Materials

The following supporting information can be downloaded, Table S1: Primer sequences used in the experiment.

## Sample Availability

Samples of the compounds are not available from the authors.
