# Supplementary material for: Different Fruit-Specific Promoters Drive AtMYB12 Expression to Improve Phenylpropanoid Accumulation in Tomato
Source: Molecules. 2022 Jan 5;27(1):317. doi: 10.3390/molecules27010317 (PMC8746655; doi:10.3390/molecules27010317)
Supplement: Supplementary file 1 [file molecules-27-00317-s001.zip › molecules-1459226-supplementary.pdf]

Table S1: Primer sequences used in the experiment

Primer name	GenBank accession	Sequence of primers
E4-F	S44898	ATGCTCGAGCCAATTCAATCTCCAATTT
E4-R	S44898	ATGACTAGTTCAAGTTCGTGTATGTTTC
2A12-F	DQ453963	ATGCTCGAGAGCACTTGTTAGACTCATCTG
2A12-R	DQ453963	ATGACTAGTAATGGTTTTGGATTAATTGC
PG-F	X07410	ATGCTCGAGCCGCATATTTAGGAGGAC
PG-R	X07410	ATGACTAGTAGAGGTATGGGAAGGGTT
E8S-F	KJ561284	ATGCTCGAGAGGAATTTCACGAAATCG
E8S-R	KJ561284	ATGACTAGTTCTTTTGCACTGTGAATG
AtMYB12-F	AT2G47460	GGACTAGTATGGGAAGAGCGCCATGTTGCG
AtMYB12-R	AT2G47460	GGACTAGTTCATGACAGAAGCCAAGCGACC
AtMYB12-qRT-F	AT2G47460	CTTCAGTCTTGTCCATCGGTG
AtMYB12-qRT-R	AT2G47460	CTAACGGTTCTCCAAAGTTCTCAC
ASR1-F	LOC543574	CCTGTTCCACCACAAGGACAA
ASR1-R	LOC543574	GTGCCAAGTTTACCGATTTGC
PAL-qRT-F	LOC112941051	AACCTATCTCGTGGCTCTTT
PAL-qRT-R	LOC112941051	TCTTTTTCGCTGAATCTTGC
C4H-qRT-F	LOC107022866	CAACAGAAAGGAGAGATCAACGAG
C4H-qRT-R	LOC107022866	CACAGCCTGAAGGTATGGAAGC
4CL-qRT-F	LOC101249624	ACACACAAAGGCTTAGTCACGA
4CL-qRT-R	LOC101249624	AACAGAGGCAACACACACATCA
CHS-qRT-F	LOC778294	TGGTCACCGTGGAGGAGTATC
CHS-qRT-R	LOC778294	GATCGTAGCTGGACCCTCTGC
CHI-qRT-F	LOC101249265	GTTTTTCACAAACCAACAGTTCTGAT
CHI-qRT-R	LOC101249265	GAAGCAGTGCTCGATTCCATAAT
F3H-qRT-F	LOC100736482	CACACCGATCCAGGAACCAT
F3H-qRT-R	LOC100736482	GCCCACCAACTTGGTCTTGTA
F3'H-qRT-F	LOC101266618	GCGATTTTATTCCGGCGCTT
F3'H-qRT-R	LOC101266618	CTGGGCCAGTATTTTCGGGT
F3'5'H-qRT-F	LOC100736504	GGCAATTGGACGAGATCCTG
F3'5'H-qRT-R	LOC100736504	AAGGAACCTCTCGGGAGTGAA
FLS-qRT-F	LOC101249699	GAGCATGAAGTTGGGCCAAT
FLS-qRT-R	LOC101249699	TGGTGGGTTGGCCTCATTAA
GT-qRT-F	LOC101260093	CGAACGACGAAACACTGTTGA
GT-qRT-R	LOC101260093	TGCAGCATAGATGGCATTGG

RT-qRT-F	LOC101244316	CTGGCAATGCAAACAGAGTGA
RT-qRT-R	LOC101244316	TCGACTTGCGGAAGAGTGAGA
C3H-qRT-F	LOC101262367	CATAAACTCTACCACCGTCTCC
C3H-qRT-R	LOC101262367	AATCCATCCCATTTCTACTCAA
HCT-qRT-F	LOC101256271	AGGTGAAAAACTCAACGATGGT
HCT-qRT-R	LOC101256271	ACACTAGGCGTGTGGAAATTAG
HQT-qRT-F	LOC544249	GTGTTTTGTTTGTTGAGGCTGA
HQT-qRT-R	LOC544249	TGATGAAGTGGATGGATGAGAG
